# Phosphorus in Beriberi and Other Diseases

**Published:** 1852-02

**Authors:** W. G. Maxwell

**Affiliations:** Garrison Surgeon, East Indies


*Phosphorus in Beriberi and other diseases.* By W. G. Maxwell, Esq., M. D., Garrison Surgeon, East Indies.—I have for several years past been trying phosphorus in beriberi, more especially in the early stages of that disease, before organic derangement sets in, and before the rapidly increasing anasarca has advanced too far.

I give the phosphorus in substance, in one, two, three, or more grains at a dose, morning or evening, or oftener, according to the urgency of the symptoms. I make the phosphorus in pills, bruising two grains or more, and then enclosing it in a wafer of dough or crumb of bread. In acute cases I make the pills fresh up at the time; but small pills, made up carefully in this way, will keep for some time. I increase or diminish the dose, as the symptoms become milder or the reverse; the urine flowing in increased quantity is the sign.

In giving the pill I make the patient keep a mouthful of water in his mouth, and the pill being dropped into it, the whole is swallowed at once. When there is want of sleep, and the bowels not fairly open, a full anodyne, with carminative tincture, together with a full purgative, all combined, are given along with the pill of phosphorus. When the motions become luminous the phosphorus can be reduced in quantity. The earlier in the disease the phosphorus is given, the more evident is action. If any tenderness of the peritoneum has set in, the phosphorus of course cannot be expected to act.

In recent cases of beriberi in young subjects, I have seen the urine increased from a few ounces to thirty pounds in the twenty-four hours after the use of phosphorus. The œdema leaves the cheeks and neck. In twenty-four hours the line can be seen across the sternum, and the patient even says he feels as if a band had been removed from his neck, and he points to the line of relief, which can be traced by the eye. These were young subjects,—eighteen to twenty-five,—and the full dose of anodyne and purgative were given at the same time.

In beriberi the motions are of an excessively green color, exactly like those passed in severe cases of recovery from cholera. It is long since I have considered beriberi and ague allied, and longer still since I could trace a relationship between ague and cholera; and if true, which I have in my works endeavored to show, the conclusion to be drawn is, that cholera and beriberi are also closely allied.—*Lancet.*

